# Zn(HQ)_2_-Phenanthroline/PEDOT:PSS Hybrid Film Engineering as a Promising Active Layer in Organic Photoconductive Devices

**DOI:** 10.3390/mi17020224

**Published:** 2026-02-08

**Authors:** María Elena Sánchez Vergara, Omar Jimenez Correa, Emilio Iván Sandoval Plata, Edgar Alvarez-Zauco, Monserrat Bizarro

**Affiliations:** 1 Faculty of Engineering, Universidad Anáhuac México, Av. Universidad Anáhuac 46, Col. Lomas Anáhuac, Huixquilucan 52786, Mexico; omar.jimenez@anahuac.mx (O.J.C.); esandoval@anahuac.mx (E.I.S.P.); 2 Fidel Velázquez Technological University, Emiliano Zapata S/N, Col. El Trafico, Nicolás Romero 54400, Mexico; 3 Faculty of Science, Universidad Nacional Autónoma de México, Circuito Exterior S/N, Ciudad Universitaria, Coyoacán, Ciudad de México 04510, Mexico; ezauco@ciencias.unam.mx; 4 Instituto de Investigaciones en Materiales, Universidad Nacional Autónoma de México, Circuito Exterior S/N, Ciudad Universitaria, Coyoacán, Ciudad de México 04510, Mexico

**Keywords:** organic semiconductor, bulk heterojunction, DFT calculation, optical properties, electrical behavior

## Abstract

Zinc(II) bis(8-hydroxyquinolinate) (Zn(HQ)_2_) and 1,10-phenanthroline (phen) were combined to fabricate an organic semiconductor in a bulk heterojunction architecture and subsequently embedded in a poly 3,4-ethylene dioxythiophene–polystyrene sulfonate (PEDOT–PSS) matrix. The resulting Zn(HQ)_2_-phen/PEDOT–PSS was deposited as a film upon tin-oxide-coated glass and graphite-covered Tetra Pak (TP)-recycled substrates for the manufacture of organic photoconductive devices. The topographical and micromechanical characteristics of the hybrid films were assessed by atomic force microscopy, with an average roughness of 5.6 nm, maximum tensile strength of 7.95 MPa, and Knoop microhardness of 14.7. The fundamental energy gap (E_g_) was determined employing the Kubelka–Munk function, with E_g_ of 3.5–3.8 eV. These results were complemented with a computational DFT molecular orbital analysis of the species involved in the hybrid semiconductor. The devices were electrically characterized under UV irradiation conditions, obtaining the current–voltage and power–voltage relationships. The maximum current in the TP–graphite device is 1.8 × 10^−2^ A and 1.1 × 10^−2^ A in the device on glass–ITO. Zn(HQ)_2_-phen/PEDOT–PSS film presents its own operating regimes relating to a photoconductor or flexible photoresistor. The power in the device on glass–ITO is 120 mW and 113 mW for shortwave and longwave, respectively, and in the device on TP–graphite, it is 198 mW and 139 mW.

## 1. Introduction

Over the last decade, semiconductor materials, such as silicon, have experienced a surge in demand, influenced by the recent COVID-19 pandemic [1] and cross-sector reliance on supply networks [2], which has led to a chip shortage. These, along with other factors, have culminated in a global semiconductor crisis [1], triggering an urge to develop new semiconductor materials at low cost, which are environmentally friendly, suitable for versatile optoelectronics applications, and manufactured with accessible infrastructures. Organic semiconductors offer a promising alternative, due to the availability and low production costs in comparison to inorganic silicon [3]. These materials, proposed as potential replacements, are macromolecular carbon-based materials known to often be π-conjugated systems in the form of polymer or small molecules [4]. Structurally, these materials may be found as thin films or molecular crystals [5]. Additionally, upon charge injection, they conduct electricity via electrodes, optical stimuli, or molecular doping, sufficient for low and flexible power devices [6], proposing silicon-free device architectures.

Hydroxyquinoline-based small molecules are recognized as good candidates to be applied as organic semiconductors due to their favorable optoelectronic properties and strong chelating ability [7]. Hydroxyquinolines are compounds formed from the incorporation of a hydroxyl group into a quinoline backbone [8], capable of binding metal ions by the bidentate chelation mechanism in which the adjacent oxygen and nitrogen atoms would bind jointly to a single metallic center, forming five-membered stable chelated rings [9]. These central atoms enhance monoprotic acidity [10], with π-conjugation from the quinoline aromatic ring [11], fluorescence, great UV-visible absorption [12], and stabilization with transition metal complexes, impacting redox behavior, including the activation of antioxidants through metal binding [13]. Due to their properties, hydroxyquinolines have gained broad application in the field of medicine, environmental sensing, and material science [10]. Such notable applications include anticancer agents, antimicrobial complexes, and neuroprotection by an iron chelator [9,14] in medicinal chemistry. Regarding material science, these compounds have a wide application that includes the first ever made organic electroluminescent diode (OLED) [15], hybrid films for organic photoconductors [16], and photonic and optical materials [17]. Specifically, 8-hydroxyquinoline (8-HQ) derivatives exhibit compound moiety, structurally containing a bicyclic compound consisting of phenol fused with a pyridine ring, where the hydroxyl group is attached in position 8. In this regard, the pyridine ring preserves the electron-deficient entity with a nitrogen property, open to presenting several structural modifications and chemical reactions due to the phenolic properties displayed by 8-HQ. These modifications encompass molecular rearrangements and electrophilic aromatic substitution. Attributable to the proximity of the heterocyclic nitrogen with the hydroxyl group, the 8-HQs are great metal ion ligands associated with the 4 and 6 covalent complexes formed via coordinated bonds. These ions include Ni^2+^, Cu^2+^, Fe^3+^, Mn^2+^, Zn^2+^, Bi^2+^, Mg^2+^, Cd^2+^, and Al^3+^ [18,19]. Although some of these metal hydroxyquinolines exhibit semiconducting behavior, they are not necessarily suitable for use in photoconductive devices. Therefore, it is necessary to study them in the presence of electron donor or acceptor molecules, in dispersed heterojunction architectures that allow for efficient charge transport across the device. In films with conventional planar heterojunction architectures, there exists a single, perfectly defined interface between the acceptor and donor molecules. In contrast, films with a dispersed heterojunction possess a mixture of the donor and acceptor molecules, resulting in a larger contact interfacial area, which can generate a greater number of charge carriers. For this reason, this type of architecture should be studied more extensively, particularly in the case of metal hydroxyquinolines.

This research aims to study the zinc(II) bis(8-hydroxyquinolinate) (Zn(HQ)_2_), in dispersion or bulk heterojunction (BHJ) with 1,10-phenanthroline (phen), for application in organic photoconductive devices. Zn(HQ)_2_ can be used as a model for organic semiconductors, finding a domain in optical waveguides, solar cells, and OLEDs, and it is presented as a great candidate for devices with high thermal stability, low operating voltage, and enhanced luminescent properties [8,20]. Phenanthroline, on the other hand, is a planar, rigid, heteroaromatic cyclic compound known to be bidentate with nitrogen ligands. They possess coordination abilities attributed to the ion pairs on both nitrogen atoms [21,22]. Furthermore, these present a three-ring and π-conjugated structure, luminescence, and electronic modification and chelating abilities [21,23,24]. These compounds have found broad application in photosensitizers for solar cells, photoelectronics, photonics, and the development of anticancer drugs [22,25]. Building on this, both phen and Zn(HQ)_2_ show great potential for applications in BHJ-based organic photoconductive devices. To study Zn(HQ)_2_-phen as an active layer, hybrid films were fabricated by embedding this BHJ in a polymer matrix of poly 3,4-ethylene dioxythiophene–polystyrene sulfonate (PEDOT–PSS). PEDOT–PSS is one of the most attractive conducting polymers for organic electronics, and it is widely accepted due to its good electrical conductivity, high optical transparency in the visible spectrum, and ease of manufacture of thin films [26,27,28,29]. It is easy to process, lightweight, and a great candidate for deposition by spin coating, ink-jet printing, and spray coating. Additionally, PEDOT–PSS presents a work function range of 4.5–5.2 eV, favorable for effective applications in photoconductive devices and suitable for use as a hole carrier layer [27,28,29,30].

## 2. Materials and Methods

All the solvents and the following reagents were obtained from a commercial source (Sigma-Aldrich, St. Louis, MO, USA): 8-hydroxyquinoline zinc (Zn(HQ)_2_: C_18_H_12_N_2_O_2_Zn), o-phenanthroline (phen: C_12_H_8_N_2_), and poly(3,4-ethylenedioxythiophene):poly(styrenesulfonate) (PEDOT–PSS: [C_8_H_8_O_3_S]_n_-[C_6_H_6_O_2_S]_n_) in H_2_O. Bulk heterojunction (BHJ) was carried out on a mixture of Zn(HQ)_2_ and phen, prepared under the mass ratio of 1:1 in the common solvent EtOH. The BHJ was performed in a heated Monowave 50 reactor (Anton Paar GmbH, Graz, Austria) operated with an integrated pressure of 20 bar for 30 min. A vacuum filtering process with EtOH cleansing and vacuum drying was performed on the reaction product, thus obtaining Zn(HQ)_2_-phen as powder. The Zn(HQ)_2_-phen was analyzed by infrared (IR) spectroscopic analysis carried out in KBr pellets with a Nicolet iS5-FT (Thermo Fisher Scientific, Inc., Waltham, MA, USA) spectrometer. Thermal stability assessment of the samples was performed with NETZSCH equipment (Erich Netzsch GmbH & Co., KG., Selb, Germany), a model Jupiter STA 449 C. Simultaneous TGA-DSC analyses were performed in argon (Ar) atmosphere from room temperature up to 650 °C, after which the Ar atmosphere was exchanged by extra dry air up to 850 °C in order to produce a degradation of the organic components. Both temperature ramps were conducted at a rate of 10 °C/min.

Zn(HQ)_2_-phen BHJ was deposited on glass slide substrates employing high-vacuum sublimation, with a mechanical pump for an initial vacuum of 10^−3^ Torr and a turbo-molecular pump for a final 10^−6^ Torr vacuum. To obtain the hybrid films, a dispersion of Zn(HQ)_2_-phen in PEDOT–PSS was prepared and mixed homogeneously with a Vortex-Genie 2 (Scientific Industries, Bohemia, NY, USA). Subsequently, to deposit the hybrid films, a 200 Smart Coater (Laurell Technologies Corporation, North Wales, PA, USA) was employed, with an angular velocity of 500 rpm for a spin time of 20 s and an acceleration of 60 rpm/s. In this experiment, 0.4 mL of dispersion was added onto the substrate, and after deposition, samples were heated at 80 °C for 3 min. The hybrid films were deposited upon the following substrates: glass, n-type silicon, as well as indium tin-oxide-coated glass slides (ITO), and recycled Tetra Pak (TP). An additional pristine PEDOT–PSS film was deposited on ITO employing the same procedure. The TP was washed with soap, water, EtOH, and MeOH. The side containing aluminum and low-density polyethylene was painted with a commercial graphitic carbon paint (composed of graphite microparticles dispersed in isopropanol and a polymer added to enhance its adherence) with a sheet resistance of 1.2 KΩ/sq. The TP-recycled pieces were air-dried overnight, and once completely dry, the TP was cut into 3 × 3 cm pieces. X-ray diffractograms were acquired with a Rigaku Ultima IV (Rigaku Corporation, Tokyo, Japan) diffractometer (CuKα, λ = 0.15418 nm). Atomic force microscopy (AFM) measurements provided insights into the topographical features, roughness, thickness, and micromechanical properties of the hybrid films, obtained with a Nanosurf Naio atomic force microscope (Nanosurf AG, Liesta, Erlen, Switzerland) with an NTEGRA platform and Gwyddion 2.66 software. The optical properties were obtained with a UV–Vis 300 Unicam (Thermo Fisher Scientific, Waltham, MA, USA) spectrophotometer and Thermo Insight software (VISIONpro). The films deposited on ITO and recycled TP allowed the fabrication of organic photoconductive devices based on a bulk heterojunction, using ITO and graphite, respectively, as an anode, and Ag as a cathode. Current–voltage (I-V) measurements of such hybrid devices were carried out with a Keithley 4200-5CS-PK1 (Tektronix, Beaverton, OR, USA) auto-ranging picometer with a four-point probe from Next Robotix (Comercializadora KMox, Mexico City, Mexico) to determine their electrical properties. In order to evaluate the I-V behavior under ultraviolet irradiation conditions, a short wavelength lamp of 254 nm and a long wavelength lamp of 365 nm UVGL-55, P/N95-0005-05/6 watt (Cambridge, CA, USA) were used.

## 3. Computational Calculations

Density functional theory (DFT) computations were performed with M06 functional [31,32], employing D3 Grimme’s dispersion [33], as implemented in the ORCA 6.1 program package [34]. The 6-31G(d,p) basis set was employed for all organic species, with a Stuttgart/Dresden pseudopotential for Zn [35]. Optimizations were performed under EtOH using the continuum solvent density mode (SMD) [36], ensuring the absence of imaginary frequencies on the vibrational analyses, for confirmation of true energy minima.

## 4. Results

**Figure 1:**
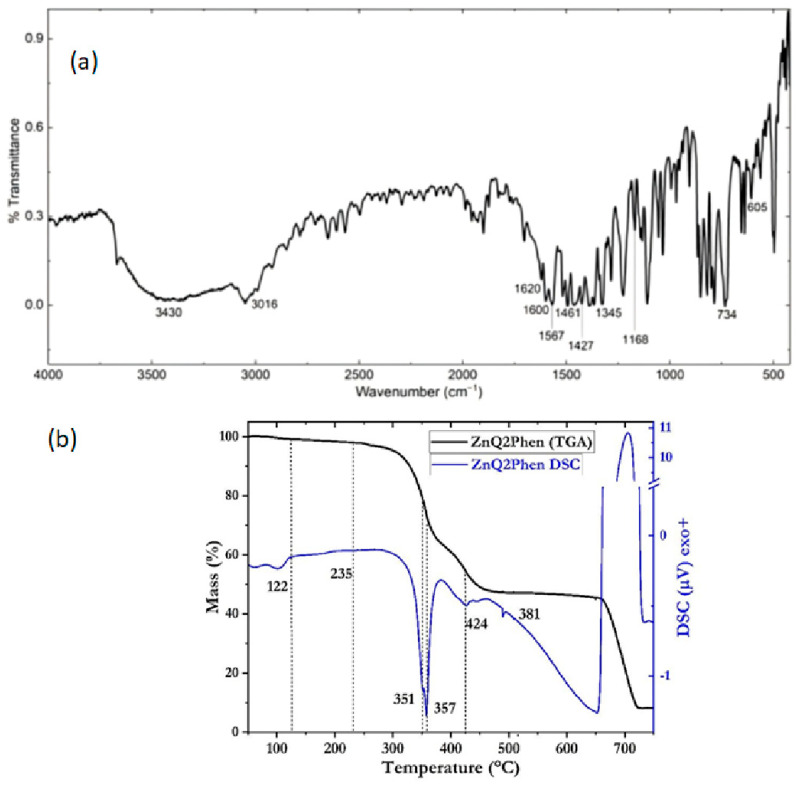
(**a**) IR spectrum and (**b**) TGA and DSC curves of the dispersed heterojunction Zn(HQ)_2_-phen BHJ.

The Zn(HQ)_2_-phen BHJ was fabricated and characterized in its main functional groups employing IR spectroscopy. Figure 1a shows the spectrum for the BHJ in KBr pellet, where signals of both hydroxyquinoline and phenanthroline are evident. Peaks appear between 400 and 600 cm^−1^ due to the stretching vibration of the zinc ion with attached ligands and the peak at 1620 cm^−1^ due to the quinoline group [37,38,39,40,41]. Also observed are the aromatic stretching C=C at 1603 cm^−1^, the C=N vibrations at 1567 cm^−1^, the C–C signal at 1461 cm^−1^, the C–C–H bending vibrations at 1168 cm^−1^, and finally, the peaks at 734 and 605 cm^−1^ are associated with in-plane ring deformations [37,38]. Regarding the presence of phenanthroline, the spectrum shows signals at 3016 cm^−1^ for C–H stretching, at 1620 and 1427 cm^−1^ corresponding to C=C bonds, and at 1345 cm^−1^ for the C–N bending vibration. During the fabrication of the Zn(HQ)_2_-phen BHJ, the solution mixture was subjected to high-pressure and temperature conditions and subsequently purified. Therefore, the IR spectroscopy results indicate that no chemical decomposition of either Zn(HQ)_2_ or phen occurred during the fabrication of the BHJ.

To study the thermal stability of Zn(HQ)_2_-phen, simultaneous TGA-DSC analyses were performed. A thermogram (Figure 1b) shows different stages where mass loss is appreciated. The first, up to 122 °C, delimited by the endothermic peak, is related to a loss of water (2%). The second one, between 235 and 460 °C, depicts the degradation of both complexes. According to Chandraleka et al. [42] and Sadeek et al. [43], the decomposition of phenanthroline occurs between 210 and 410 °C, in accordance with the endothermic phen-related peak, here located at 351 °C. Conversely, Zn(HQ)_2_ exhibits an endothermic peak at 357 °C, which is related to the fusion of Zn(HQ)_2_ with different oligomeric species of Zn(HQ)_2_ or (Zn(HQ)_2_)_4_ [44], followed by decomposition at 424 °C, related to the Zn(HQ)_2_ compound sublimation as reported by Kumar et al. [45]. In the range of 460 to 650 °C, the compound remains thermally stable. At this temperature, the extra dry air atmosphere (instead of Ar) was injected to degrade the organic components. This behavior takes place between 650 and 730 °C, with an exothermic degradation peak at 705 °C, after which, the remaining mass of 8% is attributed to the Zn contained in Zn(HQ)_2_.

**Figure 2:**
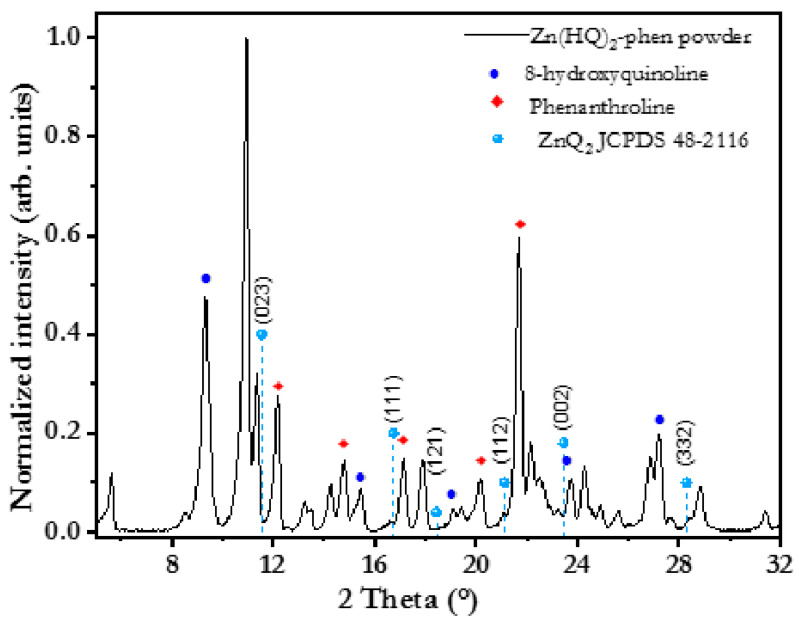
XRD pattern of Zn(HQ)_2_-phen powder sample.

XRD was performed in powder samples to analyze possible interactions between Zn(HQ)_2_ and phen. The XRD pattern (see Figure 2) shows several peaks, from which those corresponding to phen appear at 12.18, 17.08, 20.21, and 21.71°. The peaks at 11.33, 16.60, 21.1, 23.72, and 28.35° are associated with ZnQ_2_ (JCPDS 48-2116, 39-185), as reported by Shahedi et al. [46]. The presence of quinoline and phen is expected, as the compounds were mixed mechanically.

The Zn(HQ)_2_-phen BHJ was introduced into the PEDOT–PSS polymer matrix to fabricate hybrid thin films and promote BHJ–matrix charge transport within them. The increased contact interfacial area between the Zn(HQ)_2_ and phen within the BHJ and with the PEDOT–PSS creates a direct connection between each of the individual materials, favoring the diffusion of excitons, which generate the photocurrent.

**Figure 3:**
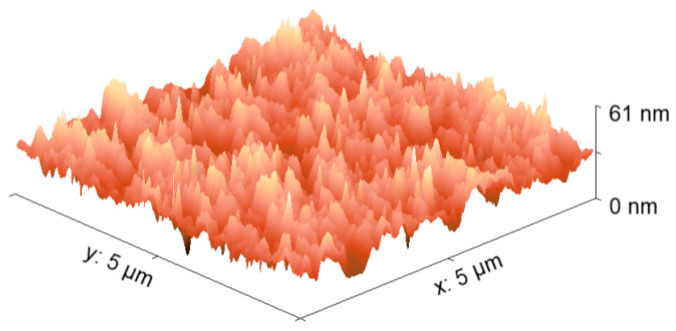
AFM image of Zn(HQ)_2_-phen/PEDOT–PSS film.

AFM was performed to analyze the topography and micromechanical properties of this hybrid film. Figure 3 shows the 3D surface image of the Zn(HQ)_2_-phen/PEDOT–PSS film on n-type silicon, used as a reference substrate. The film has a homogeneous topography that completely covers the substrate surface and exhibits a very low average roughness (Ra) of 5.6 nm and root mean roughness (RMS) of 7.1 nm.These roughness values are lower than the RMS of previously studied films with PEDOT:PSS matrix such as the graphene-PEDOT–PSS films of Hilal et al. [47], with 13.55 nm. This indicates the advantage of the BHJ over introducing nanoparticles into the hybrid film, as the inclusion of BHJ in the polymer matrix reduces the segregation between conducting PEDOT and insulating PSS, thereby reducing the electrostatic attraction between these two polymer components. In terms of micromechanical properties, the film has tensile strength (σ) of 7.95 MPa and Knoop microhardness (HK) of 14.7. Although the film’s hardness is low, its tensile strength is very high due to the PEDOT–PSS matrix, which provides greater elasticity and mechanical strength for the Zn(HQ)_2_-phen BHJ. This is advantageous when depositing the film on different substrates and fabricating various types of devices, as the mechanical strength of the films depends greatly on their composition.

**Figure 4:**
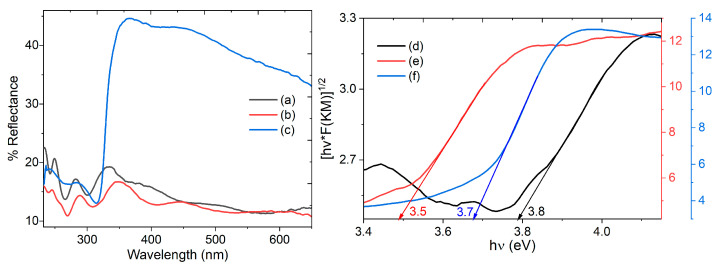
Reflectance spectrum of (a) Zn(HQ)_2_-phen on glass, (b) Zn(HQ)_2_-phen/PEDOT–PSS on glass, and (c) Zn(HQ)_2_-phen/PEDOT–PSS on TP. F(K-M) of (d) Zn(HQ)_2_-phen on glass, (e) Zn(HQ)_2_-phen/PEDOT–PSS on glass, and (f) Zn(HQ)_2_-phen/PEDOT–PSS on TP.

To experimentally determine the charge-carrying capacity of Zn(HQ)_2_-phen/PEDOT–PSS, its optical behavior was evaluated in terms of diffuse reflectance and the Kubelka–Munk function F(K–M); as if the film is to be used in photoconductive devices, it is necessary to understand its optical properties. Figure 4 presents the reflectance spectrum for the Zn(HQ)_2_-phen BHJ (Figure 4(a)) in film, as well as for the hybrid film Zn(HQ)_2_-phen/PEDOT–PSS over glass (Figure 4(b)), and TP (Figure 4(c)) substrates. It is evident that the type of substrate affects the film’s reflectance. The glass substrate BHJ and hybrid films present a similar reflectance, lower than 19% and 17%, respectively, in contrast to the film on TP, which spans up to 45%. Low reflectance is required in devices whose operation depends on absorbing as much incident light as possible to generate charge carriers; hence, the low reflectance values in glass substrate films meet this optical requirement. The Zn(HQ)_2_-phen/PEDOT–PSS hybrid film deposited on TP would, therefore, have less charge-carrying capacity. However, the 45% does not necessarily prevent it from fulfilling this function, as all the films exhibit ultraviolet absorption bands in the 200–450 nm range. The reflectance spectra are dominated by three bands, the first around 242–249 nm, the second between 281 and 288 nm, and the third around 330–370 nm. These bands are characteristic of Zn(HQ)_2_, have an intramolecular charge transfer character [48,49], and are related to a singlet exciton. They have also been reported for many other metallic hydroxyquinolines [50,51]. The bands observed at 242–249 nm and 330–370 nm can be ascribed to a charge transfer state of the Zn ion to the ligand molecule [52]. The band at 281–288 nm is related to the electronic transport between the ZnQ_2_ and the phen species.

The charge transport capacity of the hybrid film must be evaluated using its band gap. This is a fundamental parameter for semiconductors such as Zn(HQ)_2_-phen, which are intended for use as an active layer in organic devices. Therefore, the Kubelka–Munk function F(K–M), used for opaque films, was employed. This function describes how light is absorbed and scattered in a diffusely reflective material [53]. It allows the reflectance to be converted into a quantity proportional to the absorption coefficient (α) and allows the fundamental band gap (E_g_) to be calculated according to the Tauc model [54]. The equation relating F(K–M) to the Tauc expression is as follows [53,54]:

(hν × F(K-M)) = A (hν − E_g_)^P^ (1)

where h is Planck’s constant, ν is the frequency obtained from the inverse of the spectrum wavelength with the F(K-M), and A is a proportionality constant. The exponent P depends on the band structure of the semiconductor where phonon participation is required [54]. The value P = ½ is used, in this case, because, due to the amorphous nature of the hybrid film, indirect electronic transitions occur between the HOMO and the LUMO. The final procedure for calculating E_g_ consists in plotting (F(K-M))^1/2^ as a function of the photon energy (hν), linearly fitting the straight portion of the curve, and the intersection with the *x*-axis corresponding to hν provides the value of the fundamental E_g_. The F(K-M) curves of Zn(HQ)_2_-phen BHJ and the hybrid film Zn(HQ)_2_-phen/PEDOT–PSS are presented in Figure 4(d–f). The BHJ has an E_g_ of 3.8 eV, whereas Zn(HQ)_2_-phen/PEDOT–PSS exhibits an E_g_ of 3.5 eV on glass and 3.7 eV on TP substrates. The pristine BHJ exhibits a slightly larger optical band gap than when embedded in PEDOT–PSS, indicating that the presence of the polymer does not significantly alter its semiconducting behavior. The subtle variation in the optical band gap is attributed to the glass substrate promoting molecular order, generating greater intermolecular coupling and better interaction with PEDOT–PSS, which effectively reduces the HOMO–LUMO separation. In contrast, the rougher TP (polyethylene) generates greater chemical heterogeneity in the film, structural disorder, and greater electron localization, increasing the optical band gap. When comparing the E_g_ values with some hybrid films with PEDOT–PSS matrix reported in the literature, it is observed that, for instance, E. Salim et al. [55,56] made PVA/CMC/PEDOT–PSS (PCPP) polymer mixtures with different fractions of Ag nanoparticles and CMC/PVA/PEDOT–PSS/NiO, obtaining indirect optical band gaps of 4.90 eV and 4.88 eV, respectively. On the other hand, in pure PEDOT–PSS films, H. M. Ragab et al. [57] obtained indirect band gap values between 3.52 eV, and another example would be that of M.A. Morsi et al. [58] on PEO/PVA/PEDOT–PSS mixtures used as a matrix and with different fractions of CuO nanoparticles (0.4–1.6 wt%), where the indirect band gap obtained is 4.48 eV. According to these results, the Zn(HQ)_2_-phen/PEDOT–PSS film exhibits a lower band gap in most cases, and this is related to a more suitable behavior for organic semiconductors. However, it is important to consider that hybrid films have a wide band gap value, between 3.5 eV (354 nm) and 3.7 eV (335 nm), corresponding to absorption in the near-ultraviolet. Only by photon absorption in this region can they generate electron–hole pairs. Apparently, the hybrid film on glass would generate a higher photocurrent, which can be verified by the manufacture of simple devices. UV-sensitive organic photoconductors are promising for applications in selective detectors, where the visible–blind response is a key advantage, as they are engineered to selectively detect ultraviolet radiation while excluding visible light.

To determine the charge transport process in the devices, highest occupied molecular orbital (HOMO) and lowest unoccupied molecular orbital (LUMO) distributions were computationally assessed by DFT, as shown in Table 1, alongside orbital and gap energies for Zn(HQ)_2_, phen, and PEDOT–PSS. For Zn(HQ)_2_, it is evident that both HOMO and LUMO are evenly distributed over both hydroxyquinoline subunits, indicating a homogenous charge around the molecule at their frontier molecular orbitals. Nevertheless, it is possible to observe a slight distribution preference with respect to the bicyclic hydroxyquinoline systems, as HOMO is more focused around the phenolate cycles, whereas LUMO is held around the pyridine rings. Though this difference is slim, and the molecular orbitals are mostly present in the whole bis-hydroxyquinoline system, it is notable that the overall orbital distribution is absent from the Zn bridge in both HOMO and LUMO, suggesting the non-involvement of the Zn atom in the HOMO→LUMO transition. On the other hand, the overall frontier orbital distribution of phen is also evenly distributed along the molecule. HOMO appears to be horizontally spread along the full tricyclic system with π bonds. On the contrary, LUMO distribution is more focused on the lateral pyridine rings, with evident phase inversions that segment the π bonds into π*, indicating a π→π* HOMO–LUMO transition. Though the individual HOMO–LUMO theoretical gaps of Zn(HQ)_2_ and phen are not especially remarkable for organic semiconductors on their own, especially for their high LUMO energies, their heterojunction is nonetheless favorable, as closer HOMO values present ease for charge transport along the composite material. Finally, the PEDOT–PSS frontier orbital distribution was assessed by computation of an EDOT and SS tetramer oligomeric structure (EDOT)_4_(SS)_4_. HOMO spatial arrangement is completely concentrated around the (EDOT)_4_ oligomer, along the thiophene chain, with distribution distant from sulfur, and slightly toward oxygens of the ethylenedioxy rings. LUMO, on the other hand, is fully located on the (SS)_4_ oligomer, spread along the whole monomer subunit, mostly over the phenyl rings, and moderately distributed over the deprotonated sulfonic acid groups and the main polyethylene chain. Regarding the low HOMO–LUMO energy gap, it is underestimated with respect to the experimental literature, but in accordance with other DFT approximations [59]. It is, nonetheless, representative of the low band gap energy that characterizes PEDOT–PSS as a conductive polymer. Speaking of the band gap, the hydroxyquinoline molecule has greater potential as a semiconductor for charge transport than phenanthroline. However, it is the PEDOT–PSS that, according to its band gap, presents the best charge transport capacity. The band gap values are larger than those obtained for the hybrid films. From this, it can be deduced that the hybrid film exhibits better semiconducting behavior than its individual components.

**Figure 5:**
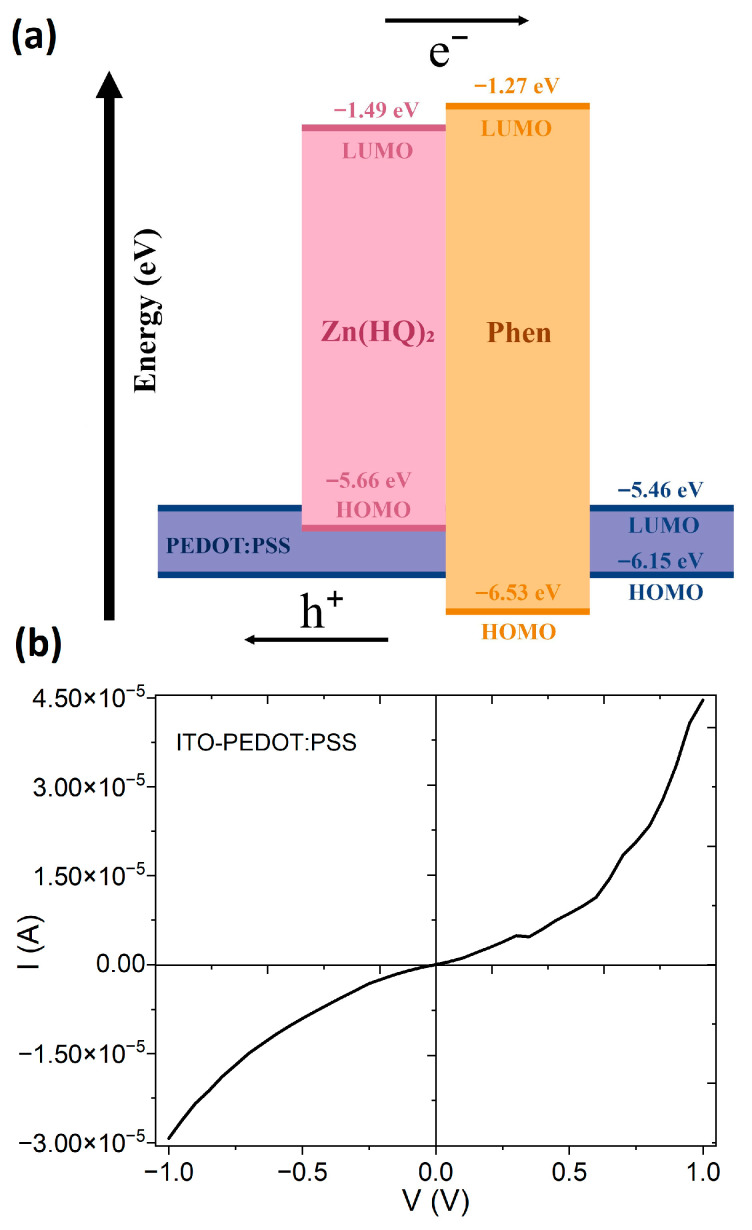
(**a**) Molecular orbital diagram of Zn(HQ)_2_-phen/PEDOT–PSS. (**b**) Current–voltage graph of the ITO/PEDOT–PSS/Ag device.

Regarding the HOMO and LUMO values obtained for each component of the hybrid film, it is important to highlight their function within photoconductive devices. Based on the orbital diagram shown in Figure 5a, it is estimated that the charge transfer mechanism within the hybrid film is generated by UV light excitation upon the hydroxyquinoline of the Zn(HQ)_2_-phen hybrid film, attributed to the higher HOMO of Zn(HQ)_2_ compared to phenanthroline, and the relative proximity of both their LUMOs. The exciton is primarily localized within Zn(HQ)_2_, but with a partial charge transfer character toward phenanthroline. Up to this point, both the electron and the hole remain Coulombically bound. With respect to LUMOs, the electron is prone to relax from the donor Zn(HQ)_2_ to the acceptor phenanthroline, creating the excited Zn(HQ)_2_^+^/phen^−^ state. This is an internal charge-transfer exciton; hence, the electrons move between phenanthroline molecules or even between neighboring Zn(HQ)_2_-phen molecules. On the other hand, when comparing the HOMOs of Zn(HQ)_2_ and phenanthroline, it is observed that the holes remain in Zn(HQ)_2_, moving between neighboring hydroxyquinoline units. Moreover, due to the polymer’s HOMO, a fraction of the holes is transferred to the PEDOT–PSS at the interface, which, within the hybrid film, creates a charge-transfer state: phen^−^/PEDOT^+^, with the electron and hole now in different domains. The holes in PEDOT–PSS move through the PEDOT lattice, while the electrons remain in phen or LUMOs of the Zn(HQ)_2_-phen complex. The system is, therefore, no longer just photoconductive Zn(HQ)_2_-phen but becomes a hybrid donor–acceptor–carrier photoconductor. Zn(HQ)_2_ acts as the hole donor, phenanthroline as the electron acceptor, and the PEDOT–PSS as the hole extractor and carrier. It is also important to consider that the PEDOT–PSS performs a double function: to provide mechanical resistance to the film and as a hole carrier layer [60]. It is important to mention that, as an initial reference, the electrical behavior current–voltage (I-V) of pristine PEDOT–PSS was evaluated, through the fabrication of the simple ITO/PEDOT–PSS/Ag device. Figure 5b shows ohmic behavior due to the conductive nature of PEDOT–PSS, resulting from the π-conjugated structure of PEDOT, which allows electron delocalization along the polymer backbone and facilitates efficient charge transport. The PSS provides charge balance and stabilizing properties [60,61]. The pristine PEDOT–PSS film presents almost ohmic contacts with electrodes Ag and ITO.

**Figure 6:**
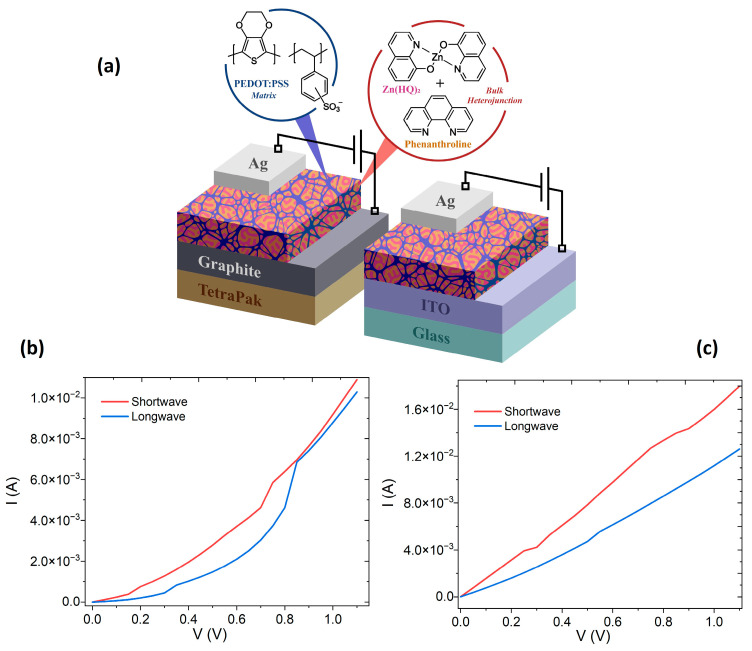
(**a**) Schematic of the Zn(HQ)_2_-phen/PEDOT–PSS device deposited on the electrode substrates: glass–ITO and TP–graphite. Device current–voltage graphs on (**b**) glass–ITO and (**c**) TP–graphite.

To evaluate the electrical behavior of the hybrid film on glass and on TP, the devices shown in Figure 6a were fabricated. The evaluation of electrical behavior was performed using the four-probe method, assuming that the bulk properties of organic semiconductors will dominate their electrical properties in the presence of a barrier to charge injection [62]. The electrical evaluation of Zn(HQ)_2_-phen/PEDOT–PSS devices was carried out under irradiation conditions with a short-wave UV lamp of 254 nm and a long-wave UV lamp of 365 nm. Figure 6b,c show the I-V behavior for the two devices, on glass/ITO and on TP/graphite. It is evident that the different lighting conditions applied to the devices affect charge transport. The I-V curves, when the glass/ITO device is irradiated, show similar behavior, with several slope changes as the voltage increases. These slope changes are due to the fact that different charge transport and injection mechanisms dominate. At low voltages, the current increases subtly; this is because electron–hole pairs are generated in the Zn(HQ)_2_-phen layer. Upon reaching 0.15 V for short-wave and 0.3 V for long-wave, a change in slope occurs that gives rise to a linear behavior where the current is dominated by the already existing photogenerated carriers and by a still very limited injection from the electrodes. As the voltage increases, the slope changes again by 0.2 V and 0.35 V for short-wave and long-wave, respectively. From these voltages, the behavior again appears exponential up to 0.7 and 0.8 V for short-wave and long-wave. This wide zone is generated because the increase in voltage helps to extract carriers trapped in Zn(HQ)_2_-phen defects and to inject new carriers from the PEDOT–PSS. The trap states of Zn(HQ)_2_-phen begin to fill, and a space-charge-limited injection (SCLC) with trap-type regime appears. Finally, from 0.75 V to 0.85 V, there is a final change in slope and an exponential behavior appears. This region occurs because, at these high voltages, the hole injection barrier from the PEDOT–PSS toward the Zn(HQ)_2_-fen is thinned, and the current increase is maintained. The greatest current transported is generated when irradiating with the short-wave lamp because it excites much more strongly, and this produces a greater amount of charge carriers, a lower interfacial barrier between the components of the hybrid film, and a faster filling of traps.

In the case of the curves of the device on TP–graphite, changes are observed with respect to those obtained for the device on glass–ITO. Although the highest transported current is also obtained with shortwave irradiation, the curves seem to have a more linear behavior. This is because the graphite electrode, due to its greater roughness with respect to the ITO, generates a dispersed contact at the interface of the Zn(HQ)_2_-phen/PEDOT–PSS film and also introduces a large series resistance that masks the internal regions of the curves. These results are interesting because with the same active Zn(HQ)_2_-phen/PEDOT–PSS film, each of the two devices presents its own operating regimes. The device on glass–ITO works under a dominant transport regime limited by volume + interface, and the hybrid film behaves as a highly sensitive organic photoconductor, while the device on TP–graphite presents the dominant regime of transport limited by contacts + substrate, and the hybrid film behaves as a flexible organic photoresistor, limited by series resistance and contacts. Another interesting aspect is that the maximum current in this device is 1.8 × 10^−2^ A, against 1.1 × 10^−2^ A in the device on glass–ITO. Table 2 shows a comparison between the two devices, with the main characteristics of each one.

**Figure 7:**
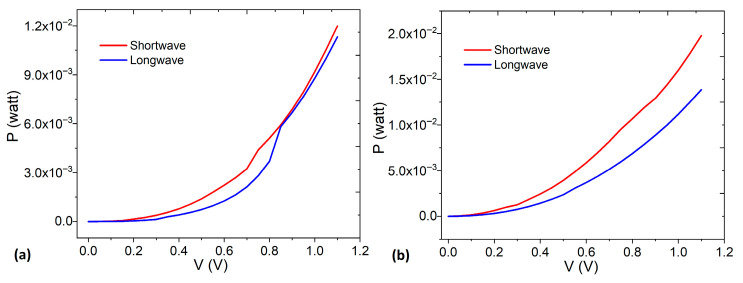
Power–voltage behavior of devices on (**a**) glass–ITO and (**b**) TP–graphite.

Finally, to determine the maximum power that the devices can deliver or consume without damage (from ambient temperature up to 60 °C), the power–voltage (P-V) behavior was analyzed. Figure 7 shows the respective curves with shortwave and longwave irradiation for the devices on glass–ITO and TP–graphite. In both cases, the power in the devices increases proportionally to the voltage, with maximum values at 1.1 V. The power in the device on glass–ITO is 120 mW and 113 mW for shortwave and longwave, respectively. Finally, the power in the device on TP–graphite is 198 mW and 139 mW for shortwave and longwave, respectively.

## 5. Conclusions

This work presented the fabrication and study of the Zn(HQ)_2_-phen/PEDOT–PSS hybrid film for its use in organic photoconductive devices. The film was fabricated by the initial mechanical preparation of the BHJ with hydroxyquinoline and phenanthroline, and subsequently by spin coating with PEDOT–PSS. The hybrid film on glass and on TP was structurally and topographically characterized by IR and AFM spectroscopy, respectively. Its tensile strength and Knoop hardness were also evaluated, yielding values of 7.95 MPa and 14.7 HK. In the film deposited on glass, the reflectance is lower than 17%, and for the film on TP, it is lower than 45%. The optical band gap on glass is 3.5 eV and on TP is 3.7 eV. Photoconductive devices were manufactured with both glass–ITO and Tetra Pak–graphite substrates used as electrodes. The devices were electrically characterized under UV irradiation conditions, and the behavior changed from a high-sensitivity organic photoconductor, limited by traps + injection + SCLC, for the case of the device on glass–ITO, to a flexible photoresistor, limited by series resistance and contacts, for the case of the device on TP–graphite.

## Figures and Tables

**Table 1:** Molecular orbitals and band gaps for Zn(HQ)_2_, phen, and PEDOT–PSS.

Molecule	HOMO	LUMO	Band Gap
Zn(HQ)_2_	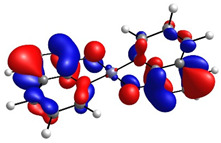 E_HOMO_ = −5.6636 eV	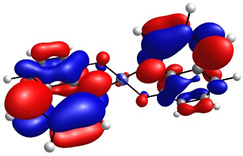 E_LUMO_ = −1.4907 eV	4.1729 eV
Phen	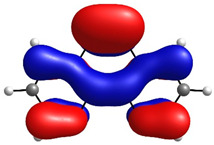 E_HOMO_ = −6.5349 eV	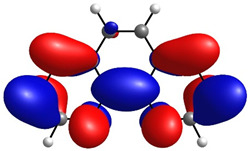 E_LUMO_ = −1.2662 eV	5.2687 eV
PEDOT–PSS	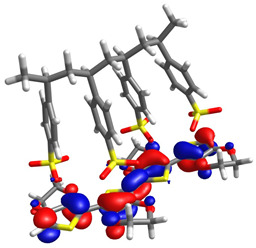 E_HOMO_ = −6.152 eV	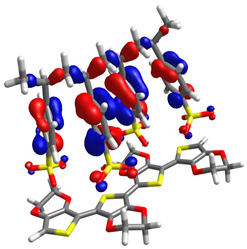 E_LUMO_ = −5.456 eV	0.696 eV

**Table 2:** Comparison of properties between Zn(HQ)_2_-phen/PEDOT–PSS devices deposited on glass–ITO and TP–graphite.

Properties	Glass–ITO	TP–Graphite
Dominant Regime	Traps + interface + SCLC	Series resistance
I-V Behavior	Nonlinear, regional behavior	ohmic-type
UV Sensitivity	High	Medium
Spectral Resolution	Better	Worse
Flexibility	No	Yes
Sustainability	Low	High

## Data Availability

The original contributions presented in this study are included in the article. Further inquiries can be directed to the corresponding author.
